# Prevalence of Parasomnias in Patients With Obstructive Sleep Apnea. A Registry-Based Cross-Sectional Study

**DOI:** 10.3389/fpsyg.2018.01140

**Published:** 2018-07-05

**Authors:** Ragnhild S. Lundetræ, Ingvild W. Saxvig, Ståle Pallesen, Harald Aurlien, Sverre Lehmann, Bjørn Bjorvatn

**Affiliations:** ^1^Department of Global Public Health and Primary Care, University of Bergen, Bergen, Norway; ^2^Norwegian Competence Center for Sleep Disorders, Haukeland University Hospital, Bergen, Norway; ^3^Department of Psychosocial Science, University of Bergen, Bergen, Norway; ^4^Department of Neurology, Haukeland University Hospital, Bergen, Norway; ^5^Department of Clinical Medicine, University of Bergen, Bergen, Norway; ^6^Department of Clinical Science, University of Bergen, Bergen, Norway

**Keywords:** parasomnia, nightmares, sleepwalking, sleep violence, sexsomnia, sleep-related eating, OSA

## Abstract

**Objective:** To assess the prevalence of parasomnias in relation to presence and severity of obstructive sleep apnea (OSA). We hypothesized higher parasomnia prevalence with higher OSA severity.

**Methods:** The sample comprised 4,372 patients referred to a Norwegian university hospital with suspicion of OSA (mean age 49.1 years, 69.8% males). OSA was diagnosed and categorized by standard respiratory polygraphy (type 3 portable monitor). The patients completed a comprehensive questionnaire prior to the sleep study, including questions about different parasomnias during the last 3 months. Pearson chi-square tests explored differences according to the presence and severity of OSA. Furthermore, logistic regression analyses with the parasomnias as dependent variables and OSA severity as predictor were conducted (adjusted for sex, age, marital status, smoking, and alcohol consumption).

**Results:** In all, 34.7% had apnea-hypopnea index (AHI) <5 (no OSA), 32.5% had AHI 5-14.9 (mild OSA), 17.4% had AHI 15-29.9 (moderate OSA), and 15.3% had AHI ≥30 (severe OSA). The overall prevalence of parasomnias was 3.3% (sleepwalking), 2.5% (sleep-related violence), 3.1% (sexual acts during sleep), 1.7% (sleep-related eating), and 43.8% (nightmares). The overall parasomnia prevalence was highest in the no OSA group. In the chi-square analyses, including all OSA groups, the prevalence of sleep-related violence and nightmares were inversely associated with OSA severity, whereas none of the other parasomnias were significantly associated with OSA severity. In adjusted logistic regression analyses the odds of sleepwalking was significantly higher in severe compared to mild OSA (*OR* = 2.0, 95% *CI* = 1.12–3.55). The other parasomnias, including sleep-related violence and nightmares, were not associated with OSA presence or severity when adjusting for sex and age.

**Conclusions:** We found no increase in parasomnias in patients with OSA compared to those not having OSA. With the exception of sleepwalking, the parasomnias were not associated with OSA severity.

## Introduction

Obstructive sleep apnea (OSA) is a sleep-related breathing disorder characterized by breathing pauses during sleep (American Academy of Sleep Medicine, 2014), with concomitant awakenings/arousals and reduction in arterial blood oxygen levels (Jordan et al., 2014). Typical symptoms are snoring and excessive daytime sleepiness (Malhotra and White, 2002). Another class of sleep disorders is parasomnias, which are defined in the International Classification of Sleep Disorders-3 (ICSD-3) as undesirable physical events or experiences that occur during entry into sleep, within sleep, or during arousal from sleep (American Academy of Sleep Medicine, 2014). The parasomnias are subdivided into three major groups; non-rapid eye movement (NREM)-related parasomnias, rapid-eye movement (REM)-related parasomnias, and other parasomnias. The first group includes disorders of arousal (confusional arousals, sleepwalking, and sleep terrors). Also, sleep-related eating disorder is classified among the NREM-related parasomnias (American Academy of Sleep Medicine, 2014). Furthermore, sleep-related violence and sexual behavior during sleep are normally classified as NREM-related parasomnias, although such behavior in some cases may arise from REM-sleep (e.g., REM-sleep behavior disorder) or reflect nocturnal seizures (Ingravallo et al., 2014). REM-related parasomnias include nightmare disorder, recurrent isolated sleep paralysis, and REM-sleep behavior disorder (American Academy of Sleep Medicine, 2014).

So far, very few studies have investigated the relationship between OSA and parasomnias. Still, according to the ICSD-3, OSA is a recognized precipitant of both sleep related abnormal sexual behaviors and disorders of arousal (American Academy of Sleep Medicine, 2014). It has been suggested that hypoxemia, caused by the apneas, can trigger confusional arousals including sexual behaviors in people with OSA (Soca et al., 2016; Kryger et al., 2017). In an epidemiological study of the US adult general population, based on 15,929 telephone interviews, people with suspected OSA were at higher risk of reporting sleepwalking episodes (Pressman, 2013). Furthermore, a similar study, including participants from Finland, Germany, Italy, Portugal, Spain and the United Kingdom (*n* = 19,961), found a significant association between sleep-related violence and breathing pauses during sleep (Ohayon and Schenck, 2010). However, both these studies suffered from a significant methodological limitation as the OSA diagnosis was based on subjective reports. Accordingly, there is need for a study where the severity of OSA can be related to the prevalence of parasomnias, using diagnostic criteria (objective registrations) for OSA.

Sleep-related eating disorder has also received focus, but much uncertainty still exists about the relation between this parasomnia and OSA. According to ICSD-3, OSA can be closely associated with sleep-related eating disorder (American Academy of Sleep Medicine, 2014). This is supported by a few studies (Eveloff and Millman, 1993; Schenck and Mahowald, 2008; Santin et al., 2014). Several cases of sleep-related violence and/or sexual behavior during sleep with forensic implications have been reported (Bornemann et al., 2006; Schenck et al., 2007; Ohayon and Schenck, 2010; Grøndahl et al., 2017). In some cases, the treating psychiatrist recognized OSA as a part of the criteria for sleepwalking (Grøndahl et al., 2017). Therefore, exploring the relationship between OSA and parasomnias is of both high scientific and forensic value.

In terms of REM-parasomnias a controlled study involving 20 patients reported that severe OSA was positively associated with nightmares (Carrasco et al., 2006). However, the evidence in the aforementioned studies is solely based on case reports or small, clinical trials (Eveloff and Millman, 1993; Carrasco et al., 2006; Schenck and Mahowald, 2008; Santin et al., 2014). The low number of participants puts severe restriction on the statistical power and the possibility to control for confounding factors known to influence both the prevalence of OSA and parasomnias, such as sex and age (Malhotra and White, 2002; American Academy of Sleep Medicine, 2014). Taken together, there is need for large-scale studies on the relationship between OSA and parasomnias, especially where the presence and severity of OSA are assessed with objective measures.

Against this backdrop, the two primary aims of the present study were: First, to assess the prevalence of parasomnias in patients with OSA and secondly, to assess the prevalence of parasomnias in relation to OSA severity in a large group of patients referred with suspicion of OSA. Our main hypotheses, based on the available literature, were that all investigated parasomnias were more prevalent in patients with OSA compared to those not having OSA, and that a dose-response relationship between OSA severity and the prevalence of parasomnias would be present.

## Materials and methods

The sample comprised 4,372 patients referred to Haukeland University Hospital with suspicion of obstructive sleep apnea between 2011 and 2016. In total, 3,050 (69.8%) of the patients were males and 1,322 (30.2%) were females. Mean age was 49.1 ± 13.5 years (range 17–89).

All patients underwent a standard respiratory polygraphic sleep study using a type 3 portable monitor (Embletta™ or NOX T3, Resmed Norway AS) as described previously (Bjorvatn et al., 2015). The vast majority of the patients were recorded while sleeping at home, while the rest of the patients slept in a hospital hotel. Scoring rules were in accordance with the 2007 American Academy of Sleep Medicine manual (Iber et al., 2007), defining apneas as a reduction of 90% or more of baseline nasal airflow with a duration of at least 10 s. Hypopneas were defined as a nasal flow reduction of 30–90% of baseline, lasting at least 10 s accompanied by an oxygen desaturation of ≥ 4%. Obstructive sleep apnea was diagnosed and classified in accordance with the apnea-hypopnea index (AHI): No OSA (AHI < 5); mild OSA (AHI 5-14.9); moderate OSA (AHI 15-29.9); severe OSA (AHI ≥ 30).

The patients completed a comprehensive questionnaire prior to the respiratory sleep study, covering many aspects of sleep medicine. A nurse and/or doctor revised the questionnaire during a consultation to make sure the answers were reliable. Adjustments were made if necessary. The present study focused on the items related to parasomnias. Participants were asked if they had experienced sleepwalking, sleep related violence, sexual acts during sleep, sleep-related eating or nightmares (see Supplementary Material for the parasomnia questionnaire). The time frame was last 3 months, and the questions had four response alternatives (yes, often; yes, sometimes; never; don't know). In the statistical analyses, the parasomnia variables were dichotomized into yes (including “yes, often” and “yes, sometimes”) and no (including “never” and “don't know”). Items regarding marital status, dichotomized into married/cohabiting and living alone, and current smoking habits and alcohol consumption were also included in the analysis. The smoking variable was dichotomized into non-smoking and smoking daily (including 1 or more cigarettes per day). For alcohol consumption the respondents were asked about frequency of such consumption. A response format with five alternatives (never; rarely; 1–2 days per week; 3–5 days per week; daily) was used, and the variable was dichotomized into <3 days per week and ≥3 days per week.

### Ethics

The study was approved by The Regional Committee for Medical and Health Research Ethics, Health Region West (REC 2014/1060). Written informed consent was obtained from all patients.

### Statistics

IBM SPSS Statistics, version 24.0 was used for the data analyses. Differences in characteristics of patients categorized according to the presence and severity of OSA were explored using Pearson chi-square tests (with Yates' correction for continuity when used in a 2 × 2 table). Furthermore, separate logistic regression analyses were conducted with the different parasomnias as dependent variables and OSA severity (mild, moderate, severe) as predictor. Significant predictors from the chi-square analyses were entered as co-variates; the first analysis adjusted for sex and age, whereas the second analysis adjusted for sex, age, marital status, smoking and alcohol. Significance level was set to 0.05.

## Results

Among the patients referred to the hospital with suspicion of obstructive sleep apnea, AHI < 5 was found in 34.7%, 32.5% had mild OSA, 17.4% had moderate OSA, and 15.3% had severe OSA (Table 1), respectively. Thus, the prevalence rate of OSA (AHI ≥ 5) in the present study population was 65.2%. OSA severity was positively associated with male sex (Table 1). Table 1 also shows that age and alcohol consumption were positively associated with OSA severity, whereas smoking was negatively associated with OSA severity.

**Table 1:** Characteristics of patients referred to a university hospital with suspicion of obstructive sleep apnea.

	**Apnea-hypopnea index (AHI) categories**					
	**No OSA AHI < 5 % (*n*)**	**Mild OSA AHI 5–14.9 % (*n*)**	**Moderate OSA AHI 15–29.9 % (*n*)**	**Severe OSA AHI ≥ 30 % (*n*)**	**Chi-square (df)^a^**	***p*-value^b^**
Patients	34.7 (1,469)	32.5 (1,377)	17.4 (736)	15.3 (649)		
Sex, males	60.7 (891)	69.1 (951)	77.2 (568)	84.0 (545)	139.6 (3)	<**0.0005**
**AGE^c^**						
17–30 years 31–40 years 41–50 years 51–60 years >60 years	73.0 (330) 50.8 (337) 35.2 (396) 23.6 (257) 16.5 (149)	19.0 (86) 29.4 (195) 34.8 (391) 36.1 (393) 34.6 (312)	4.2 (19) 9.3 (62) 16.0 (180) 22.5 (245) 25.5 (230)	3.8 (19) 10.5 (70) 14.0 (157) 17.7 (193) 23.5 (212)	615.0 (12)	<**0.0005**
Marital status, married/cohabiting	75.4 (1,067)	75.4 (1,014)	75.5 (547)	78.1 (489)	2.1 (3)	0.55
Daily smoking	25.2 (347)	22.2 (286)	18.7 (131)	19.7 (118)	14.4 (3)	**0.002**
**ALCOHOL^c^**						
< 3 days per week ≥3 days per week	35.6 (1,355) 23.4 (75)	32.8 (1,249) 30.0 (96)	16.8 (639) 26.3 (84)	14.9 (568) 20.3 (65)	34.0 (12)	<**0.0005**
Sleepwalking	4.1 (59)	2.5 (34)	2.6 (19)	4.0 (25)	7.3 (3)	0.06
Sleep-related violence	3.3 (48)	2.2 (30)	1.7 (12)	1.4 (9)	10.0 (3)	**0.02**
Sexual acts during sleep	3.8 (55)	2.6 (35)	3.5 (25)	2.1 (13)	6.5 (3)	0.09
Sleep-related eating	1.9 (27)	1.6 (21)	1.0 (7)	1.6 (10)	2.6 (3)	0.45
Nightmares	50.1 (718)	42.5 (574)	40.1 (289)	36.9 (232)	40.5 (3)	<**0.0005**

a Degrees of freedom.

b Pearson chi-square.

c *The distribution of AHI categories is presented for each group within age/alcohol. Significant findings are shown in **bold***.

In the chi-square analyses, including all OSA groups, the prevalence of sleep-related violence and nightmares were inversely associated with OSA severity, whereas none of the other parasomnias were significantly associated with OSA severity (Table 1). When dichotomizing the AHI categories into “no OSA” (AHI < 5) and “OSA” (AHI ≥ 5), the prevalence of sleepwalking (4.1 vs. 2.9%, *p* = 0.042), sleep-related violence (3.3 vs. 1.9%, *p* = 0.005) and nightmares (50.1 vs. 40.6%, *p* < 0.005) were significantly higher in the no OSA group. In contrast, the chi-square tests did not show any significant difference between the “no OSA” and the “OSA” groups in terms of sexual acts during sleep (3.8 vs. 2.7%, *p* = 0.053) and sleep-related eating (1.9 vs. 1.4%, *p* = 0.292).

Table 2 presents the prevalence of the different parasomnias in the study population. Nightmare was the most common parasomnia, with 6.5% answering “yes, often” and 37.3% answering “yes, sometimes.” Furthermore, 3.3% of the patients had been sleepwalking often or sometimes during the last 3 months, 2.5% had injured themselves or somebody else in their sleep, 3.1% had performed sexual acts during sleep, and 1.6% had eaten food during sleep (Table 2).

**Table 2:** Prevalence of parasomnias in patients referred with suspicion of obstructive sleep apnea.

	**Experienced parasomnia during the last three months % (*****n*****)**			
	**Yes, often % *(n)***	**Yes, sometimes % *(n)***	**No % *(n)***	**Don't know % *(n)***
Sleepwalking	0.7 (32)	2.6 (110)	92.6 (3,963)	4.1 (176)
Sleep-related violence	0.1 (3)	2.4 (103)	96.5 (4,132)	1.0 (42)
Sexual acts during sleep	0.3 (14)	2.8 (121)	93.1 (3,980)	3.7 (159)
Sleep-related eating	0.4 (16)	1.3 (54)	97.0 (4,147)	1.4 (60)
Nightmares	6.5 (279)	37.3 (1,594)	49.9 (2,132)	6.2 (264)

Male sex was positively associated with both sleepwalking and sexual acts during sleep (Table 3). In contrast, males had lower prevalence of nightmares than females. All parasomnias, except for sleep-related eating, were negatively associated with age; that is, the younger age the higher parasomnia prevalence (Table 3). Furthermore, patients living alone reported higher prevalence of sleep-related eating and nightmares. Smoking was positively associated with all the parasomnias except sleep-related violence, whereas increased alcohol consumption was only (and positively) associated with sleepwalking.

**Table 3:** Prevalence of parasomnias within sex, age, marital status, smoking, and alcohol in patients referred with suspicion of obstructive sleep apnea.

	**Sleepwalking**		**Sleep-related violence**		**Sexual acts during sleep**		**Sleep-related eating**		**Nightmares**	
	**% (n)**	**Chi-square^a^ (df) *p-value***	***% (n)***	**Chi-square^a^ (df) *p-value***	**% (n)**	**Chi-square^a^ (df) *p-value***	***% (n)***	**Chi-square^a^ (df) *p-value***	***% (n)***	**Chi-square^a^ (df) *p-value***
**SEX**										
Female Male	2.4 (31) 3.7 (111)	4.5 (1) ***p** = **0.034***	1.8 (23) 2.8 (83)	3.2 (1) *p = 0.072*	1.6 (20) 3.9 (115)	14.8 (1) ***p**<**0.0005***	1.5 (19) 1.7 (51)	0.2 (1) *p = 0.678*	51.5 (655) 40.8 (1218)	38.0 (1) ***p**<**0.0005***
**AGE**										
17–30 years 31–40 years 41–50 years 51–60 years >60 years	5.4 (25) 5.3 (35) 3.3 (37) 2.6 (29) 1.7 (16)	23.1 (4) ***p**<**0.0005***	8.0 (37) 2.9 (19) 2.0 (23) 1.3 (14) 1.4 (13)	70.6 (4) ***p**<**0.0005***	7.5 (35) 4.3 (28) 2.7 (31) 3.1 (34) 0.8 (7)	49.3 (4) ***p**<**0.0005***	2.6 (12) 2.0 (13) 1.4 (16) 1.8 (20) 1.0 (9)	6.0 (4) *p = 0.199*	63.3 (295) 54.0 (356) 42.0 (477) 39.1 (431) 34.6 (314)	142.3 (4) ***p**<**0.0005***
**MARITAL STATUS**										
Married/cohabiting Living alone	3.3 (105) 3.1 (32)	0.0 (1) *p = 0.863*	2.5 (79) 2.3 (23)	0.0 (1) *p = 0.763*	3.0 (96) 3.5 (36)	0.5 (1) *p = 0.485*	1.3 (41) 2.6 (26)	7.0 (1) ***p** = **0.005***	40.5 (1281) 54.1 (550)	57.9 (1) ***p**<**0.0005***
**SMOKING**										
No Yes	2.6 (82) 5.3 (48)	15.3 (1) ***p**<**0.0005***	2.3 (72) 3.3 (30)	2.9 (1) *p = 0.112*	2.7 (85) 4.9 (44)	9.9 (1) ***p** = **0.002***	1.3 (40) 3.0 (27)	11.6 (1) ***p** = **0.001***	41.8 (1309) 52.2 (474)	30.7 (1) ***p**<**0.0005***
**ALCOHOL**										
< 3 days per week ≥ 3 days per week	3.1(122) 5.2 (17)	4.0 (1) ***p** = **0.045***	2.4 (93) 3.4 (11)	1.2 (1) *p = 0.273*	3.2 (123) 3.4 (11)	(1) *p = 0.841*	1.6 (63) 1.8 (6)	(1) *p = 0.772*	44.1 (1714) 41.4 (135)	0.9 (1) *p = 0.348*

a *Pearson Chi-Square was used for age and alcohol. Yates' Correction for Continuity was used for the 2 x 2 tables (sex, marital status, and smoking). Significant findings are shown in **bold***.

Table 4 shows the results from the logistic regression analyses with different parasomnias as dependent variables. In the analyses adjusted for sex and age we found no significant associations between OSA severity (mild OSA as reference) and the parasomnias. However, when adjusting for sex, age, marital status, smoking and alcohol, we found that patients with severe OSA were two times more likely to report sleepwalking than patients with mild OSA. When performing similar logistic regression analyses with “no OSA” as reference, we found no significant association between OSA and any of the parasomnias (data not shown).

**Table 4:** Logistic regression analyses with different parasomnias as dependent variables among patients with obstructive sleep apnea.

	**Sleepwalking**		**Sleep-related violence**		**Sexual acts during sleep**		**Sleep-related eating**		**Nightmares**	
	**Adjusted OR^a^ (95% CI) *n* = 2,710**	**Adjusted OR^b^ (95% CI) *n* = 2,505**	**Adjusted OR^a^ (95% CI) *n* = 2,708**	**Adjusted OR^b^ (95% CI) *n* = 2,503**	**Adjusted OR^a^ (95% CI) *n* = 2,705**	**Adjusted OR^b^ (95% CI) *n* = 2,501**	**Adjusted OR^a^ (95% CI) *n* = 2,706**	**Adjusted OR^b^ (95% CI) *n* = 2,502**	**Adjusted OR^a^ (95% CI) *n* = 2,700**	**Adjusted OR^b^ (95% CI) *n* = 2,496**
Mild OSA (AHI 5-14.9)	1.00	1.00	1.00	1.00	1.00	1.00	1.00	1.00	1.00	1.00
Moderate OSA (AHI 15-29.9)	1.13 (0.64–2.02)	1.16 (0.62–2.17)	0.81 (0.41–1.61)	0.76 (0.36–1.60)	1.47 (0.86–2.51)	1.68 (0.96–2.93)	0.60 (0.25–1.42)	0.61 (0.25-1.48)	1.02 (0.85-1.24)	1.00 (0.82-1.21)
Severe OSA (AHI ≥ 30)	1.65 (0.96-2–83)	**2.00 (1.12–3.55)**	0.67 (0.31–1.44)	0.76 (0.35–1.67)	0.82 (0.43–1.59)	0.81 (0.40–1.65)	0.97 (0.45–2.10)	0.92 (0.41–2.07)	0.91 (0.74–1.11)	0.91 (0.74–1.13)

a Adjusted for sex, age.

b *Adjusted for sex, age, marital status, smoking, and alcohol. OR, odds ratio. CI, confidence interval. Significant values are shown in **bold***.

## Discussion

In this large scale study on patients referred to a hospital on suspicion of OSA, we found no significant increase in the prevalence of parasomnias in the patients with OSA compared to those not having OSA. In fact, the prevalence of all parasomnias was highest in the group with AHI < 5 (albeit not significant for sexual acts during sleep and sleep-related eating). These findings were in contrast with our initial hypothesis of all the investigated parasomnias being more prevalent in patients with OSA compared to those not having OSA. Our second hypothesis, that there would be a dose-response relationship between OSA severity and the prevalence of parasomnias was partly confirmed, as the prevalence of sleepwalking was higher in patients with severe OSA compared to those with mild OSA, when adjusted for several confounders. However, OSA severity was not associated with the other parasomnias.

The available literature has reported possible associations between parasomnias and OSA. In the present study, it was therefore surprising to find that the prevalence of all parasomnias was highest in the no OSA group. However, NREM-related parasomnias occur in connection with slow wave sleep (Bjorvatn et al., 2010) which often is reduced in patients with OSA (Verma et al., 2001). Hence, based on this sleep characteristic, one could possibly expect less parasomnias with more apneas during the night. This notion was supported by the results from the chi-square analyses, where sleep-related violence was found to be negatively associated with OSA severity. However, most parasomnias are known to decrease with age (American Academy of Sleep Medicine, 2014), and a possible explanation for the finding of no increase in parasomnias with higher OSA severity might be that the severe OSA group was significantly older than the no OSA group. When adjusting for sex and age in the logistic regression analyses, the decrease in sleep-related violence with OSA severity did not remain significant. Furthermore, none of the other parasomnias were associated with OSA severity, when adjusting for sex and age. In the fully adjusted model (for sex, age, marital status, smoking, and alcohol) only sleepwalking was associated with OSA severity, although sleep-related violence, sexual acts during sleep and sleep-related eating are related to sleepwalking (Schenck et al., 2007; Ohayon and Schenck, 2010; Santin et al., 2014). The reason for these inconsistent findings is not clear, but the association between OSA severity and sleepwalking may reflect that OSA is a unique trigger of sleepwalking specifically, e.g., through a mechanism such a sleep density being reinforced by respiratory events (Espa et al., 2002). Future studies should therefore investigate if OSA is a trigger for specific NREM-related parasomnias.

In addition, there was a tendency of a dose-response relationship between OSA severity and the prevalence of sleepwalking also when adjusting only for sex and age, supporting the notion about an association between the two sleep disorders. Contrary to expectations, we found no clear dose-response relationship between OSA severity and the prevalence of the other parasomnias in the largest study of this kind up to now.

Several studies have aimed to investigate the prevalence of parasomnias in the general population. Bjorvatn et al. found that the current prevalence of sleepwalking in the Norwegian adult population was 1.7% (Bjorvatn et al., 2010). Similar studies from the USA and the UK reported a prevalence of sleepwalking among adults ranging from 1 to 2% (Ohayon et al., 1999; Pressman, 2013). Similarly, the prevalence of sleep-related violence is reported to be 1.6–2.1% (Ohayon et al., 1997; Ohayon and Schenck, 2010). In contrast, we found that 4.1% of the patients in the no OSA group reported sleepwalking, and that 3.3 and 3.8% reported sleep-related violence and sexual acts during sleep, respectively. Furthermore, the prevalence of nightmares (50.1% in the no OSA group) was much higher than what has been reported in the general population (19.4%) (Bjorvatn et al., 2010). The only parasomnia in our study that was comparable to other studies with respect to prevalence was sleep-related eating (Bjorvatn et al., 2010). Importantly, the group with no OSA can on no account be compared to the general population. Since all patients were referred to a hospital with suspicion of OSA, this means that they all had symptoms or complaints, i.e., snoring, breathing pauses during sleep, tiredness, etc. of OSA. This may explain why we found a higher prevalence of e.g., sleepwalking and nightmares in patients with no OSA, compared to what is reported in the general population. The high prevalence of parasomnias may be related to negative affectivity, as previous research has found that individuals high in negative affectivity tend to report lower sleep quality than individuals low in negative affectivity (Fortunato and Harsh, 2006). Patients having problems with their sleep may report having nightmares more frequently because they associate having bad sleep with having bad dreams. Similar to other studies (Bjorvatn et al., 2010; American Academy of Sleep Medicine, 2014), women reported more nightmares than men. Furthermore, male sex was associated with greater OSA severity. The observed decrease in nightmares with OSA severity in the chi-square analyses could thus be attributed to sex, as the severe OSA group had 23.3% less women than the no OSA group. Accordingly, when adjusted for sex and age, the prevalence of nightmares was no longer significantly associated with OSA severity.

Some strengths and limitations of the present study should be noted. An obvious strength was the large sample, making it possible to adjust for relevant confounding factors. Furthermore, the vast majority (>90%) of the patients referred to the hospital with suspicion of OSA agreed to provide data, resulting in a representative sample of patients referred to OSA assessment. Another important asset of the present study was the use of objective measures of AHI, eliminating the influence from the common method bias, when analyzing these data against self-reports provided by the patients (Podsakoff et al., 2003). One limitation was that we used polygraphy, and not polysomnography, in diagnosing OSA. This may pose a problem as AHI is reported to be underestimated in polygraphic registrations compared to polysomnography (Masa et al., 2011; Nerfeldt et al., 2014). Furthermore, the lack of polysomnography recordings prevents distinction between sleep-related violence from NREM-sleep and from REM sleep-behavior disorder (RBD). RBD is the only parasomnia that includes polysomnography in the diagnostic criteria, while all other parasomnias can be diagnosed based on subjective reports (American Academy of Sleep Medicine, 2014). We cannot be sure that sleepwalking, sleep-related violence, sexual acts during sleep, and sleep-related eating reported in the present study occurred during NREM or REM-sleep. However, if one of these parasomnias occurs with RBD in the same patient, both should be diagnosed (American Academy of Sleep Medicine, 2014). This has been referred to as parasomnia overlap disorder. Hence, a similar study using polysomnography should be performed. The current investigation was limited by not using a validated questionnaire to assess the prevalence of parasomnias. Furthermore, the presence of parasomnias was self-reported and may thus likely be underestimated. However, we adjusted for marital status, probably minimizing this effect as individuals living alone can generally be expected to be more unaware of parasomnias than those being in a relationship (Ohayon and Schenck, 2010). The AHI is referring to both obstructive and central apneas. As the AHI is also increased in central sleep apnea (American Academy of Sleep Medicine, 2014), this may be a source of error if the fraction of central apneas is high. However, the percentage of patients with mainly central apneas in the clinic is very low, thus we have no reason to believe that lack of differentiation between obstructive and central apneas have made any major impact on the reported findings. Another limitation concerns the fact that all patients were studied for one night only, hence the results may have been influenced by the first night effects. Since the vast majority of the patients were recorded while sleeping at home, this effect was probably minimized (Edinger et al., 1997).

In conclusion, we found no significant increase in parasomnias in patients with OSA compared to those not having OSA. Sleepwalking was however found to be more commonly reported by patients with severe OSA compared to patients with mild OSA, even after adjusting for relevant confounders like sex, age, marital status, smoking, and alcohol consumption. The finding that parasomnias, with exception of sleepwalking, were not associated with OSA severity was somewhat unexpected as it is not in line with previous research and theoretical notions. This emphasizes the need for more large-scale studies using objective measurements for diagnosing OSA to further elucidate the relationship between OSA and different parasomnias.

## Author contributions

All authors listed have made a substantial, direct and intellectual contribution to the work, and approved it for publication.

### Conflict of interest statement

The authors declare that the research was conducted in the absence of any commercial or financial relationships that could be construed as a potential conflict of interest.
