# Supplementary material for: Prevalence of Parasomnias in Patients With Obstructive Sleep Apnea. A Registry-Based Cross-Sectional Study
Source: Front Psychol. 2018 Jul 5;9:1140. doi: 10.3389/fpsyg.2018.01140 (PMC6042013; doi:10.3389/fpsyg.2018.01140)
Supplement: Supplementary file 1 [file Table_1.DOCX]

***Supplementary material***

**Prevalence of parasomnias in patients with obstructive sleep apnea. A registry-based cross-sectional study**

**Ragnhild S. Lundetræ^1*^, Ingvild W. Saxvig^2^, Ståle Pallesen^2,3^, Harald Aurlien^4,5^, Sverre Lehmann^2,6^, and Bjørn Bjorvatn^1,2^**

^1^ Department of Global Public Health and Primary Care, University of Bergen, Bergen, Norway.

^2^ Norwegian Competence Center for Sleep Disorders, Haukeland University Hospital, Bergen, Norway.

^3^ Department of Psychosocial Science, University of Bergen, Bergen, Norway.

^4^ Department of Neurology, Haukeland University Hospital, Bergen, Norway.

^5^ Department of Clinical Medicine, University of Bergen, Bergen, Norway

^6^ Department of Clinical Science, University of Bergen, Bergen, Norway.

*Correspondence: Ragnhild S. Lundetræ: ragnhild.lundetre@student.uib.no

**Supplementary Table 1. Parasomnia questions.**

| 1. During the last three months, have you been sleepwalking? |
| --- |
| 1. During the last three months, have you injured yourself or somebody else in your sleep? |
| 1. During the last three months, have you performed sexual acts in your sleep? |
| 1. During the last three months, have you eaten food in your sleep? |
| 1. During the last three months, have you had nightmares? |

Scale: yes, often; yes, sometimes; no; don’t know
